# Recurrent Metastatic Basal Cell Carcinomas of the Face in a Patient with Gorlin–Goltz Syndrome

**DOI:** 10.3390/curroncol32040193

**Published:** 2025-03-26

**Authors:** Petko Petrov, Dobromira Shopova, Georgi Goranov, Atanaska Dinkova, Nina Stoyanova, Nikolay Yanev

**Affiliations:** 1 Department of Maxillofacial Surgery, Faculty of Dental Medicine, Medical University-Plovdiv, 4000 Plovdiv, Bulgaria; 2 Department of Prosthetic Dentistry, Faculty of Dental Medicine, Medical University-Plovdiv, 4000 Plovdiv, Bulgaria; 3 Section Cardiology, First Department of Internal Diseases, Medical University-Plovdiv, 4000 Plovdiv, Bulgaria; georgi.goranov@mu-plovdiv.bg; 4 Department of Oral Surgery, Faculty of Dental Medicine, Medical University-Plovdiv, 4000 Plovdiv, Bulgaria; atanaska.dinkova@mu-plovdiv.bg; 5 Department of Ophthalmology, Faculty of Medicine, Medical University-Plovdiv, 4000 Plovdiv, Bulgaria; nina.stoyanova@mu-plovdiv.bg; 6 Department of Medical and Clinical-Diagnostic Activities, Ruse University “Angel Kanchev”, 7000 Ruse, Bulgaria; nyanev@abv.bg

**Keywords:** Gorlin–Goltz syndrome (GGS), basal cell carcinoma syndrome (NBCCS), basal cell carcinomas (BCCs), metastatic basal cell carcinomas (BCCs)

## Abstract

Gorlin–Goltz syndrome, also known as nevoid basal cell carcinoma syndrome (NBCCS), is a rare, inherited autosomal dominant disorder primarily caused by mutations in the *PTCH1* gene, which regulates the Hedgehog signaling pathway. This genetic defect leads to the uncontrolled proliferation of basal cells, resulting in the formation of multiple basal cell carcinomas (BCCs) and odontogenic keratocysts (OKCs). This study aims to present a complex clinical case of a patient with Gorlin–Goltz syndrome who developed multiple recurrent metastatic basal cell carcinomas on the facial region, detailing the multidisciplinary treatment strategies employed and the challenges encountered during the management of the disease. The patient, diagnosed with a pathogenic *PTCH1* gene mutation, underwent a series of treatment interventions over several years. These included multiple surgical excisions aimed at tumor removal, diverse radiotherapy approaches for residual or inoperable lesions, and systemic targeted therapy with Hedgehog pathway inhibitors to control tumor progression. The recurrent and aggressive nature of the basal cell carcinomas resulted in extensive facial tissue loss, posing significant challenges for radical tumor excision and subsequent reconstructive procedures. Multimodal therapeutic strategies, including Mohs micrographic surgery for precise tumor clearance and targeted systemic therapy with vismodegib, were implemented. However, the aggressive progression of lesions required ongoing surgical interventions, highlighting the limitations of current treatment modalities in achieving long-term disease control. This case underscores the critical need for a comprehensive, multidisciplinary approach to managing Gorlin–Goltz syndrome. Successful management requires close collaboration between dermatologists, oncologists, maxillofacial surgeons, and plastic surgeons to balance effective tumor control with optimal functional and aesthetic outcomes. The integration of advanced surgical techniques and targeted molecular therapies shows promise in improving patient outcomes. Nonetheless, early diagnosis, rigorous follow-up, and patient education remain essential components in minimizing disease progression and enhancing the quality of life for affected individuals.

## 1. Introduction

Gorlin–Goltz syndrome, also known as nevoid basal cell carcinoma syndrome (NBCCS), is a rare autosomal dominant genetic disorder characterized by the presence of multiple basal cell carcinomas (BCCs), odontogenic keratocysts, and developmental anomalies affecting the skeletal system and other organs [1,2,3]. The syndrome is most commonly caused by mutations in the *PTCH1* gene, which encodes a protein involved in the Hedgehog signaling pathway—a critical regulator of cellular growth and development [4,5,6].

Gorlin–Goltz syndrome presents with several hallmark clinical manifestations: (i) cutaneous manifestations: multiple basal cell carcinomas that may develop at a young age [7]; (ii) odontogenic keratocysts: jaw cysts that can result in facial deformities and dental complications [8]; (iii) skeletal abnormalities: Including bifid ribs, scoliosis, and other bone malformations [9]; (iv) neurological and ophthalmological manifestations: such as calcification of brain structures and ocular anomalies [10].

The diagnosis is established based on clinical criteria, which require either the presence of two major criteria or one major and two minor criteria [11]. The major diagnostic criteria include the following: multiple basal cell carcinomas or at least one BCC before the age of 20; histologically confirmed odontogenic keratocysts; palmar or plantar pits (three or more); calcification of the falx cerebri; bifid, fused, or markedly broadened ribs [12,13,14]. Early identification and multidisciplinary management of Gorlin–Goltz syndrome are essential to mitigate complications and improve patient outcomes.

The management of Gorlin–Goltz syndrome (Nevoid Basal Cell Carcinoma Syndrome) is inherently multidisciplinary, focusing on symptom control, complication prevention, and consistent monitoring for the early detection of new lesions or tumors [15,16]. Although no specific cure exists for the underlying genetic mutation, treatment strategies are tailored to address the syndrome’s diverse manifestations. Surgical excision remains the most commonly used method for removing basal cell carcinomas (BCCs), providing effective tumor elimination with minimal recurrence risk [17]. For larger lesions or those located in cosmetically sensitive areas, Mohs micrographic surgery is preferred due to its tissue-sparing approach that ensures complete tumor removal while preserving healthy surrounding skin [17]. Non-surgical therapies also play a vital role, particularly for patients with multiple or superficial lesions. Topical treatments, such as Imiquimod cream and 5-Fluorouracil cream, are effective for managing small, surface-level BCCs [18]. Additionally, photodynamic therapy (PDT) offers a non-invasive option for treating multiple superficial lesions by using photosensitizing agents activated by light to destroy cancerous cells [19].

Systemic therapies, particularly Hedgehog pathway inhibitors like vismodegib and sonidegib, have shown significant effectiveness in patients with multiple or inoperable BCCs. These medications target the defective Hedgehog signaling pathway, addressing the molecular mechanism responsible for tumor development in Gorlin–Goltz syndrome [20]. Beyond active treatments, preventive and supportive care is essential. Regular dermatological screenings allow for early detection and timely management of new skin lesions, while dental and maxillofacial monitoring helps identify and manage odontogenic keratocysts [15]. Orthopedic and neurological evaluations are necessary to monitor and address skeletal and neurological abnormalities that may arise [16]. Moreover, strict sun protection measures, including the use of broad-spectrum sunscreens, protective clothing, and avoidance of prolonged sun exposure, are crucial in reducing the risk of skin cancer development [15]. A personalized and comprehensive treatment plan that integrates surgical, medical, and preventive strategies is essential for effectively managing Gorlin–Goltz syndrome and improving the patient’s long-term quality of life.

Management of odontogenic keratocystic tumors in Gorlin–Goltz syndrome involves localized excision of cysts using minimally invasive methods. Curettage or marsupialization is often performed to prevent recurrences, given the high relapse rate of these lesions. Regular dental examinations are essential for the early detection and timely management of new cyst formations [12].

Treatment of skeletal deformities focuses on correcting scoliosis or other bone malformations when they cause functional impairments. In severe cases, orthopedic interventions may be necessary to improve mobility and quality of life [14].

Medulloblastoma, particularly in pediatric patients, requires vigilant monitoring through regular brain imaging (MRI or CT scans) to enable early detection. If a tumor is diagnosed, treatment may include surgical resection, radiotherapy, or chemotherapy. Additionally, ongoing monitoring for intracranial calcifications and neurological symptoms is critical for comprehensive care [21].

Genetic counseling is recommended for patients and their families to assess the risk of disease inheritance and provide guidance on preventive strategies. Sun exposure should be minimized, and the consistent use of broad-spectrum sunscreens (SPF 50+) is strongly advised to reduce the risk of developing basal cell carcinomas [22].

Psychological support, including psychotherapy, is important for helping patients cope with the emotional stress associated with managing a chronic condition. Participation in patient support groups can also provide valuable emotional and social support for individuals with Gorlin–Goltz syndrome [23].

Routine dermatological evaluations every 6–12 months are necessary for the early detection of new skin lesions. Imaging studies (X-rays, MRI) are vital for monitoring skeletal abnormalities and potential neurological complications. Regular dental check-ups are also important for the timely identification of new cysts [24].

With early diagnosis and appropriate multidisciplinary management, patients with Gorlin–Goltz syndrome can maintain a good quality of life. However, continuous medical surveillance is crucial due to the persistent risk of recurrences and complications [25,26]. If this syndrome is suspected, consultation with a geneticist or a specialist in rare diseases is recommended for accurate diagnosis and tailored treatment guidance.

In this context, we present a case of a patient with Gorlin–Goltz syndrome who developed multiple facial basal cell carcinomas. Despite prior surgical interventions, in the various forms of radiotherapy, and targeted therapy, the facial lesions continued to recur, posing significant challenges in therapeutic management

## 2. Case Report

A 43-year-old male patient presented with multiple postoperative scars on the skin of the face, neck, and back. He was diagnosed with a genetic mutation in the *PTCH1* gene. The disease manifested in early adolescence, with the initial presentation being the development of numerous small tumors on the facial skin. His first surgical intervention was performed in 2007, where histological verification confirmed basal cell carcinoma. In his history of the disease, postoperative radiotherapy was administered as part of the treatment, which is contraindicated for patients diagnosed with Gorlin syndrome.

The patient sought help from our team at the University Hospital “MEDIKA” Ruse, Clinic of Maxillofacial Surgery in April 2023, after a recurrence of the formation on the left neck. He had previously been treated in other centers, and the information presented regarding previous treatments was collected from his medical records. In 2018, cervical swelling was noted, and contrast-enhanced computed tomography (CT) revealed a cluster of lymph nodes. Local excision was performed, confirming metastasis from basal cell carcinoma. A follow-up positron emission tomography (PET) scan in 2019 showed a metabolically active lesion involving the left cervical lymph nodes and a solitary bone metastasis in the occipital region. Palliative radiotherapy was administered to the left neck and supraclavicular region, delivering a cumulative dose of 30 Gy in November 2019. Targeted therapy with Erivedge (vismodegib), 150 mg daily, was initiated. Additionally, localized focused radiotherapy was applied to the bone lesion. Topical treatment with Aldara (imiquimod), an immune response modifier, was used on a solitary lesion of the scalp, located in the right frontoparietal region. He has been taking Vismodegib since November 2019. During the period of taking this medication, a delayed appearance of new lesions and recurrence of old ones was observed. Vismodegib was interrupted only during the period of taking Cisplatin, for about two months. Another option could be Cemiplimab. In other European countries, the USA, etc., Cemiplimab and Pembrolizumab are licensed for use in basal cell carcinoma, but in Bulgaria, there is currently no such authorization. It is not yet licensed for use for basal cell carcinomas, is not included in the basal cell carcinoma treatment protocols, and is not paid for by the Health Insurance Fund.

A follow-up CT scan in May 2020 revealed a lesion in the upper third of the left sternocleidomastoid muscle. Surgical excision confirmed histological metastasis from basal cell carcinoma. The patient underwent five cycles of chemotherapy with monotherapy cisplatin and received adjuvant radiotherapy. Control CT scans in 2021 indicated a stable condition without disease progression. However, a CT scan in May 2022 showed progression of the cervical lymph nodes on the left side. A decision was made to proceed with stereotactic surgery, followed six months later by CyberKnife treatment.

**Figure 1:**
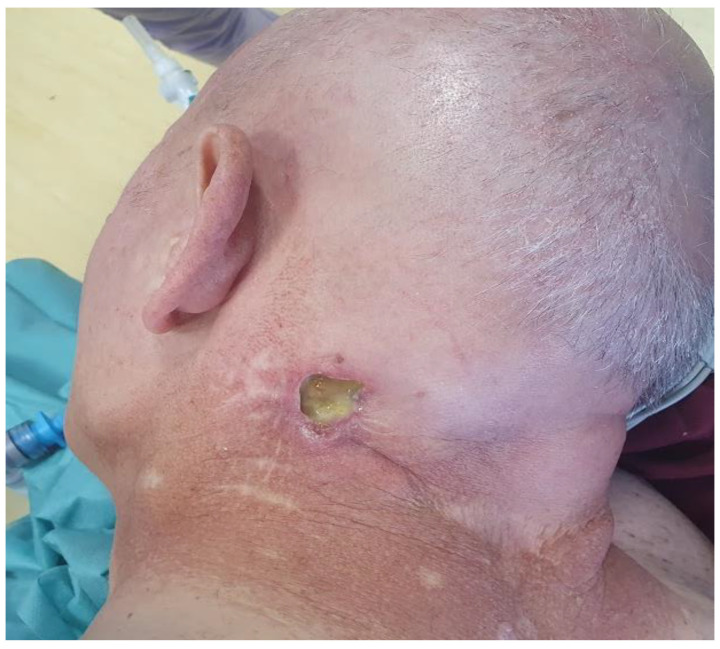
Status of the cervical lesion after CyberKnife treatment, May 2023.

**Figure 2:**
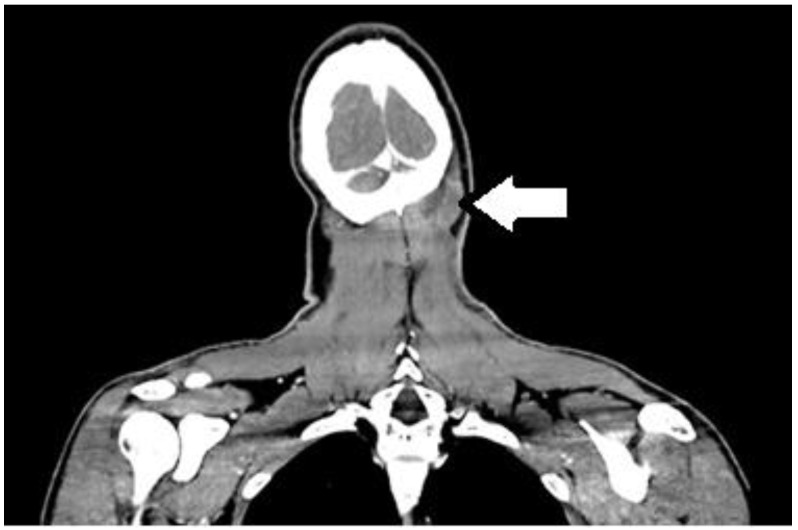
Massive infiltrate occipitally and paravertebral on the left, May 2023.

One month post-procedure, the cervical lesion began to ulcerate, forming a non-healing, secreting ulcer in the right nuchal region, measuring approximately 2 cm in diameter (Figure 1). Contrast-enhanced CT imaging revealed a massive infiltrate in the underlying musculature (Figure 2).

**Figure 3:**
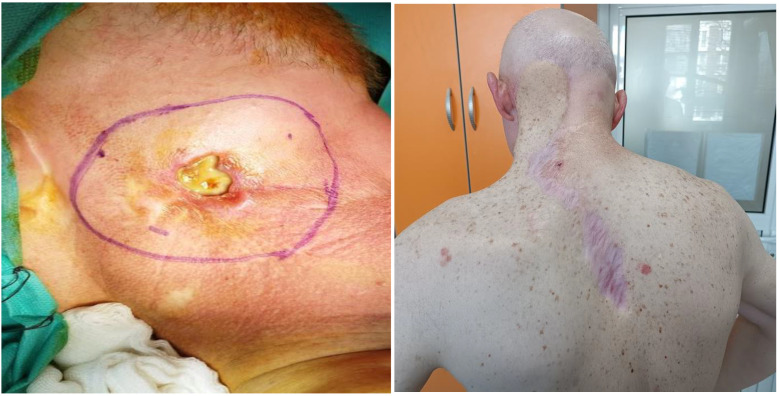
Block resection with single-stage plastic surgery.

A decision was made for en bloc excision of the lesion and single-stage reconstruction with a trapezoidal flap (Figure 3).

**Figure 4:**
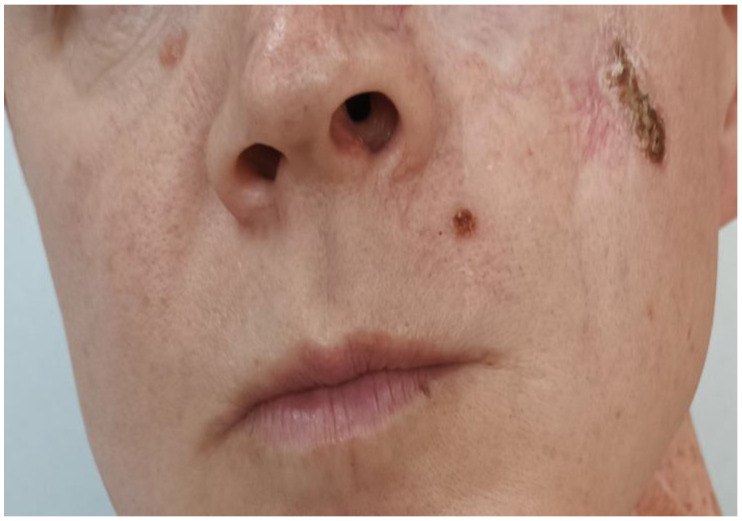
Newly appeared lesions on the face.

The resection margins were clear of tumor and a year later, on follow-up CT and MRI, there were no signs of local recurrence in the intervention area. However, newly appearing lesions were recorded infraorbital and on the upper lip on the left (Figure 4).

**Figure 5:**
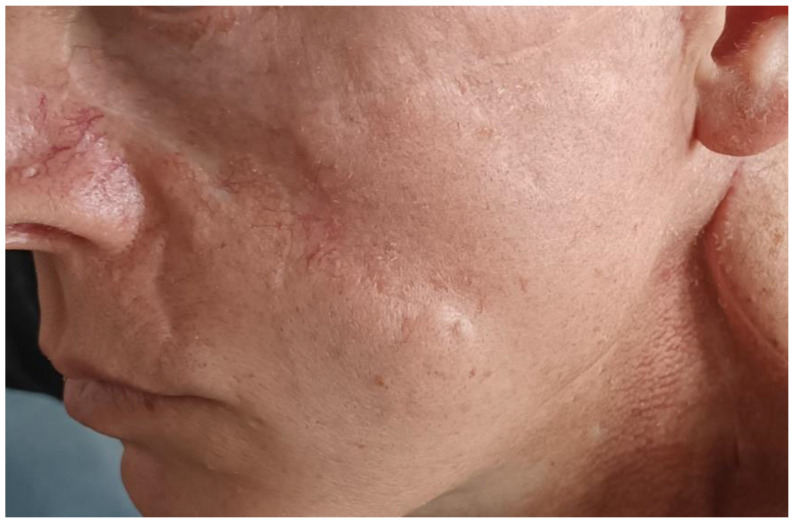
No evidence of local recurrence, with respect to the excised facial lesions.

In August 2024, local excision and one-step Mustarde plastic surgery were performed (Figure 5).

**Figure 6:**
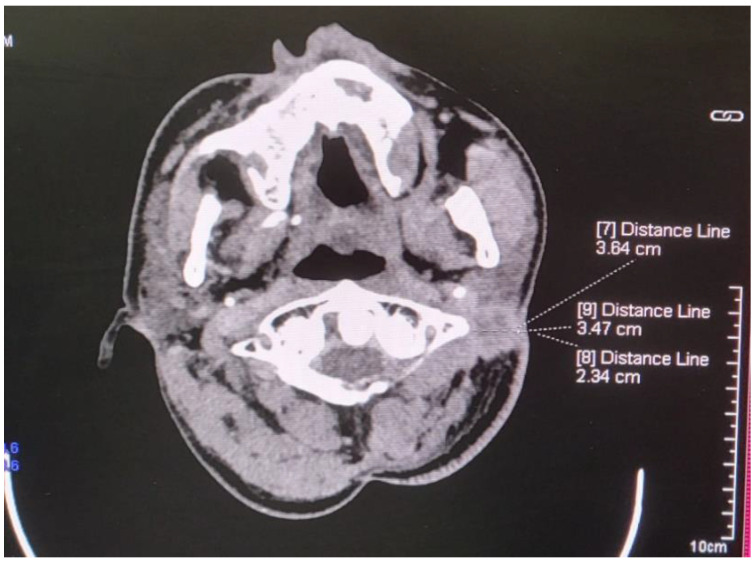
Suspected metastatic lesion on the neck.

In November 2024, new, suspicious metastatic lymph nodes at the second cervical level are visualized on a control CT scan (Figure 6).

Cytological and histological verification and subsequent presentation to the oncology committee are pending.

## 3. Discussion

Recurrences of basal cell carcinomas (BCCs) are common in patients with Gorlin–Goltz syndrome due to the genetic basis of the disease. The primary cause is a mutation in the *PTCH1* gene, leading to continuous activation of the Hedgehog signaling pathway, which predisposes individuals to the development of new BCCs [3,4,5,15].

The causes of basal cell carcinoma recurrence include genetic predisposition, sun exposure, incomplete tumor resection, and multiple primary basal cell carcinomas Patients with Gorlin–Goltz syndrome have a significantly increased risk of developing multiple BCCs even after successful treatment of initial lesions. The defect in the cellular repair mechanisms results in the accumulation of additional mutations, facilitating the formation of new tumors [22,27]. Ultraviolet (UV) radiation is a major contributing factor to the development of new lesions and the recurrence of existing ones. Patients are particularly vulnerable to UV-induced DNA damage due to their impaired genetic repair systems [28]. Incomplete removal of malignant cells during initial surgical intervention can lead to local recurrence. Surgical margins that are too narrow or have residual microscopic disease can contribute to tumor regrowth [29]. Multiple primary basal cell carcinoma patients with Gorlin–Goltz syndrome frequently develop new primary tumors, which can be mistaken for recurrences. The continuous formation of independent BCCs complicates the differentiation between true recurrences and newly developed lesions [30].

Effective prevention strategies are essential for reducing the risk of recurrence in patients with Gorlin–Goltz syndrome. These strategies focus on regular monitoring, protection from ultraviolet (UV) radiation, pharmacological interventions, and optimizing surgical techniques. Dermatological examinations every 3–6 months are crucial for early detection and timely treatment of new basal cell carcinomas. Regular monitoring helps in identifying suspicious lesions before they progress, enabling prompt intervention [31]. Consistent use of broad-spectrum sunscreens with SPF 50+ to protect against harmful UV rays. Wearing protective clothing, wide-brimmed hats, and UV-blocking sunglasses to minimize sun exposure. Avoiding direct sunlight, especially between 10:00 a.m. and 4:00 p.m., when UV radiation is at its peak [28]. Hedgehog pathway inhibitors (e.g., vismodegib, sonidegib) are used to prevent recurrences and manage multiple basal cell carcinomas by targeting the defective signaling pathway [18,32]. Topical medications such as imiquimod or 5-fluorouracil are effective for treating superficial lesions, offering a non-invasive therapeutic option [2,6]. Mohs micrographic surgery is recommended for its precision in removing cancerous tissue while preserving healthy skin, significantly reducing the risk of recurrence. This technique ensures complete tumor excision with minimal damage to surrounding tissue, making it particularly suitable for lesions in cosmetically sensitive areas [5,6,33].

The approach to recurrences of basal cell carcinomas is appropriate to be multidisciplinary, after reevaluation and confirmed diagnosis and personalized treatment plan. Biopsy is essential for confirming the recurrence of basal cell carcinomas. This helps to ensure an accurate diagnosis and determine the appropriate course of action for subsequent treatment [34]. A multidisciplinary team involving dermatologists, oncologists, and geneticists is crucial for the optimal management of recurrences. Collaborative decision-making ensures a comprehensive treatment plan, considering both the clinical and genetic aspects of the disease [35,36]. Tailored therapy is selected based on the size, location, and type of the recurring tumor. Treatment options include the following: surgical removal for well-defined, localized recurrences; photodynamic therapy for superficial lesions or those in cosmetically sensitive areas; targeted therapy, particularly with Hedgehog pathway inhibitors, for larger or multiple recurrent tumors that are not amenable to surgery [37,38,39].

The Italian Association of Medical Oncologists (AIOM), utilizing the Grading of Recommendations, Assessment, Development, and Evaluation (GRADE) approach, provides clinical guidance for the diagnosis, treatment, and monitoring of patients with cutaneous squamous cell carcinoma (cSCC). Traditionally, chemotherapy and targeted therapy have been the standard treatment for advanced cSCC, but the recent approval of immunotherapeutic agents, such as Cemiplimab and Pembrolizumab, has significantly expanded treatment options [40]. Similarly, for patients with advanced basal cell carcinoma (BCC) who are not candidates for curative surgery or radiotherapy, Hedgehog pathway inhibitors (HHIs) such as vismodegib and sonidegib have been established as first-line systemic therapies [41]. These HHIs, along with the PD-1 checkpoint inhibitor Cemiplimab, have been approved by the European Medicines Agency (EMA) for advanced BCC, offering new therapeutic possibilities [42]. While immunotherapy can lead to durable responses in some patients, a substantial proportion may not respond or may develop resistance [2]. Cemiplimab, in particular, has demonstrated clinically meaningful antitumor activity and an acceptable safety profile in patients with metastatic BCC who have experienced disease progression or intolerance to HHI therapy [43]. Additionally, it is the first immune checkpoint inhibitor approved by the U.S. Food and Drug Administration (FDA) for refractory BCC, marking a significant advancement in immunotherapy for this disease [44]. Despite these developments, there remains a clinical need for effective systemic therapies for advanced synchronous BCC/cSCC that are not amenable to local treatments. Given the increasing global life expectancy, cases of advanced synchronous BCC/cSCC are expected to become more frequent, highlighting the necessity for further investigation into the synergistic potential of targeted therapies and immunotherapy, whether administered in combination or sequentially [42]. In Bulgaria, Cemiplimab is approved for use in squamous cell carcinoma, but not yet for basal cell carcinoma.

This personalized approach helps maximize treatment effectiveness while minimizing potential risks and complications.

Patients with Gorlin–Goltz syndrome must be informed about the high risk of recurrences and the necessity for continuous care to minimize complications and ensure a good quality of life. This includes regular follow-ups, proactive prevention strategies, and a comprehensive approach to managing both the skin and internal manifestations of the syndrome. Proper patient education is essential for enhancing adherence to treatment plans and improving long-term outcomes.

## 4. Conclusions

Gorlin–Goltz syndrome is a rare but significant genetic disorder that requires a multidisciplinary approach to diagnosis and treatment. The main features include multiple basal cell carcinomas, keratocystic odontogenic tumors, skeletal abnormalities, and an increased risk of medulloblastoma and other tumors.

Thanks to modern diagnostic methods and advances in treatment, such as targeted therapies with Hedgehog inhibitors, it is possible to significantly improve the prognosis and quality of life for patients. However, early diagnosis, regular follow-up, and interdisciplinary collaboration among specialists are crucial for the effective management of the disease.

Genetic counseling also plays a key role, in enabling awareness and prevention for future generations. Support from family, social networks, and the medical team is essential for the successful adaptation to life with the syndrome.

## Data Availability

Data are contained within the article.

## Abbreviations

GGS	Gorlin–Goltz syndrome
NBCCS	basal cell carcinoma syndrome
BCCs	basal cell carcinomas
